# Image-Guided Stereotactic Body Radiotherapy (SBRT) with Enhanced Visualization of Tumor and Hepatic Parenchyma in Patients with Primary and Metastatic Liver Malignancies

**DOI:** 10.3390/cancers17071088

**Published:** 2025-03-25

**Authors:** Alexander V. Kirichenko, Danny Lee, Patrick Wagner, Seungjong Oh, Hannah Lee, Daniel Pavord, Parisa Shamsesfandabadi, Allen Chen, Lorenzo Machado, Mark Bunker, Angela Sanguino, Chirag Shah, Tadahiro Uemura

**Affiliations:** 1 Division of Radiation Oncology, Allegheny Health Network Cancer Institute, Pittsburgh, PA 15212, USA; danny.lee@ahn.org (D.L.); seungjong@wustl.edu (S.O.); hlee@mhpdoctor.com (H.L.); daniel.pavord@ahn.org (D.P.); parisa.shamsesfandabadi@ahn.org (P.S.); chirag.shah@ahn.org (C.S.); 2 Division of Surgical Oncology, Allegheny Health Network Cancer Institute, Pittsburgh, PA 15212, USA; patrick.wagner@ahn.org; 3 Division of Abdominal Transplantation, Allegheny General Hospital, Pittsburgh, PA 15212, USA; allenchen@ahn.org (A.C.); lorenzomachado@ahn.org (L.M.); tadahiro.uemura@ahn.org (T.U.); 4 Department of Clinical Pathology, Allegheny General Hospital, Pittsburgh, PA 15212, USA; mark.bunker@ahn.org (M.B.); angela.sanguino@ahn.org (A.S.)

**Keywords:** SPION, MR-Linac, liver metastases, hepatocellular carcinoma, adaptive radiotherapy, liver parenchyma sparing, SBRT, local control, bridge to transplant

## Abstract

Goal: This study evaluates the feasibility and outcome of a personalized MRI-based liver SBRT treatment planning platform with the SPION contrast agent Ferumoxytol^®^ (Sandoz Inc.; Princeton, NJ, USA) to maintain a superior real-time visualization of liver tumors and volumes of functional hepatic parenchyma for radiotherapy planning throughout multi-fractionated liver SBRT with online plan adaptations on an Elekta Unity 1.5 T MR-Linac (Elekta; Stockholm, Sweden). Materials and Methods: Patients underwent SPION-enhanced MRI on the Elekta Unity MR-Linac for improved tumor and functional hepatic parenchyma visualization. An automated contouring algorithm was applied for the delineation and subsequent guided avoidance of functional liver parenchyma volumes (FLVs) on the SPION-enhanced MR-Linac. Radiation dose constraints were adapted exclusively to FLV. Local control, toxicity, and survival were assessed with at least 6-month radiographic follow-up. Pre- and post-transplant outcomes were analyzed in the subset of patients with HCC and hepatic cirrhosis who completed SBRT as a bridge to liver transplant. Model of End-Stage Liver Disease (MELD-Na) was used to score hepatic function before and after SBRT. Results: With a median follow-up of 23 months (range: 3–40 months), 23 HCC patients (26 lesions treated) and 9 patients (14 lesions treated) with hepatic metastases received SBRT (mean dose: 48 Gy, range: 36–54 Gy) in 1–5 fractions. Nearly all patients in this study had pe-existing liver conditions, including hepatic cirrhosis (23), prior TACE (7), prior SBRT (18), or history of hepatic resection (2). Compared to the non-contrast images, SPIONs improved tumor visibility on post-SPION images on the background of negatively enhancing functionally active hepatic parenchyma. Prolonged SPION-contrast retention within hepatic parenchyma enabled per-fraction treatment adaptation throughout the entire multi-fraction treatment course. FLV loss (53%, *p* < 0.0001) was observed in cirrhotic patients, but functional and anatomic liver volumes remained consistent in non-cirrhotic patients. Mean dose to FLV was maintained within the liver threshold tolerance to radiation in all patients after the optimization of Step-and-Shoot Intensity-Modulated Radiotherapy (SS-IMRT) on the SPION-enhanced MRI-Linac. No radiation-induced liver disease was observed within 6 months post-SBRT, and the MELD-Na score in cirrhotic patients was not significantly elevated at 3-month intervals after SBRT completion. Conclusions: SPION Ferumoxytol^®^ administered intravenously as an alternative MRI contrast agent on the day of SBRT planning produces a long-lasting contrast effect between tumors and functional hepatic parenchyma for precision targeting and guided avoidance during the entire course of liver SBRT, enabling fast and accurate online plan adaptation on the 1.5 T Elekta Unity MR-Linac. This approach demonstrates a safe and effective bridging therapy for patients with hepatic cirrhosis, leading to low toxicity and favorable transplant outcomes.

## Simple Summary

The reliable identification of liver tumors and functional hepatic parenchyma on a hybrid MR-Linac system has a direct impact on SBRT planning and treatment outcomes. This study evaluates the impact of the superparamagnetic iron oxide nanoparticle (SPION) alternative contrast agent Ferumoxytol^®^ on tumor visibility and functional liver mapping during per-fraction adaptive radiation therapy using the 1.5 T Elekta Unity MR-Linac system. Our findings demonstrate that SPION-enhanced MRI provides consistent tumor visualization throughout the entire SBRT course, thereby facilitating precise tumor targeting and the conformal avoidance of functional liver parenchyma. This novel approach enables a more individualized treatment strategy, particularly beneficial for patients with hepatic cirrhosis and hepatocellular carcinoma requiring SBRT as bridging therapy prior to liver transplantation.

## 1. Introduction

The integration of magnetic resonance imaging (MRI)-Linac hybrid technology with MRI-guided radiotherapy (MRgRT) has revolutionized radiotherapy planning and delivery, offering superior soft-tissue contrast and real-time tumor visualization compared to conventional CT-guided radiotherapy [1,2]. A recent investigation into the feasibility of MRgRT for treating primary liver malignancies demonstrated in-field local control rates of 75–100% at 1 year, with a median SBRT dose of 50 Gy (30–50 Gy) delivered in five fractions [3,4,5]. Moreover, MRI during liver SBRT provides the advantage of the real-time delineation of anatomical changes in tumors and normal tissues throughout treatment. This enables per-fraction treatment plan optimization, substantially reducing treatment-related toxicities with MRgRT, potentially enhancing the therapeutic ratio [6]. 

**Figure 1:**
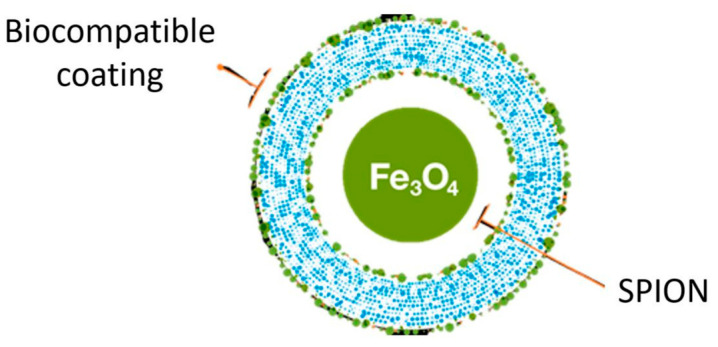
Pictorial presentation of superparamagnetic iron oxide nanoparticle (SPION) agent Ferumoxytol^®^.

In this context, the reliable identification of liver tumors and functional hepatic parenchyma in hybrid MR-Linac imaging has a direct impact on radiotherapy planning and treatment outcomes. Hepatic metastases, and especially HCC lesions, exhibit significant variability and inconsistency in terms of MR imaging characteristics, related to the cirrhotic liver background and parenchymal heterogeneity [7,8]. Therefore, acquiring contrast-enhanced imaging is crucial for the rapid and accurate delineation of tumors and organs at risk during online treatment plan adaptations. Liver-specific contrast agents, such as gadoxetic acid (GdEOB-DTPA or Gd-BOPTA), have previously been reported in the context of online MRgRT [9]. However, repeated administration within a short time frame raises concerns about potential renal toxicity. Ferumoxytol^®^ (Sandoz Inc.; Princeton, NJ), an ultra-small superparamagnetic iron oxide nanoparticle (SPION) agent, has been approved by the U.S. Food and Drug Administration (FDA) for intravenous iron replacement therapy in patients with chronic kidney disease [10]. However, it is increasingly being investigated and utilized as an alternative contrast agent for liver MRI [11,12,13,14,15,16,17]. It consists of a superparamagnetic Fe_3_O_4_ iron oxide core coated with a layer of dextran, a semi-synthetic carbohydrate that protects bioactive iron from interactions with plasma components (Figure 1). 

Within minutes of injection, SPIONs are rapidly absorbed by mononuclear macrophages in the reticuloendothelial system (RES). Over time, they are gradually released into the iron metabolic pathway as the dextran undergoes enzymatic degradation [17]. SPIONs, when sequestered in the deep cellular compartments of hepatic Kupffer cells (KC), generate strong and uniform T2, as well as T2* relaxation effects within the liver parenchyma infiltrated by KCs. Malignant tumors, which lack KCs, show no signal change, thereby enhancing tumor-to-liver contrast and improving MR detection [13,16,17]. Once cleared from the bloodstream, SPIONs remain within the KCs of liver parenchyma for up to two months [15,16]. This makes SPIONs particularly attractive as MR contrast agents, providing a consistent visualization of liver malignancies and functional hepatic parenchyma throughout the duration of MRI-guided adaptive liver SBRT on MR-Linac systems. 

Due to its unique metabolic and magnetic properties, the off-label use of Ferumoxytol has gained popularity as an alternative MRI contrast agent with significant safety advantages: (a) it can be safely administered to patients with impaired renal function for whom gadolinium-based MRI contrast agents are contraindicated; (b) unlike gadolinium-containing agents, Ferumoxytol is not associated with the risk of long-term accumulation, such as brain deposition from repeated use [11,12,13].

SPIONs have been utilized for MRgRT on a 0.35 T MR-Linac [18,19], but have not yet been applied to the high-field 1.5 T Elekta Unity MR-Linac. The primary objective of our study was to evaluate the visualization of liver tumors and functional liver parenchyma during treatment planning using SPION-enhanced 1.5 T MRI and per-fraction MR image sets. Additionally, our study aimed to (1) evaluate toxicity, local control, and survival outcomes following liver SBRT on a SPION-enhanced MRI-Linac and (2) assess clinical and pathologic outcomes in a subgroup of liver-transplant-eligible patients with hepatic cirrhosis who completed liver SBRT using SPION-enhanced MRI. 

## 2. Methods

### 2.1. Patients and Study Design

Between June 2021 and September 2024, a total of 32 patients, including 23 patients (26 treated lesions) with isolated HCC and 9 patients (14 treated lesions) with isolated liver metastases from colorectal (6) or breast cancers (3), were enrolled in this institutional review board-approved prospective study with informed consent for the off-label use of Ferumoxytol^®^ as an MRI contrast agent. 

Median follow-up was 23 months (range, 3–40 months). Nearly all patients in this study had prior liver-directed therapies, including patients with hepatic metastases and prior liver SBRT (6), hepatic resection (2), or combination of both (4). Patients with hepatocellular carcinoma had Child–Pugh A (5) or B (18) cirrhosis and a mean MELD-Na score of 13.7 (range, 8–21). Seven patients in the HCC subgroup had completed prior TACE or prior liver SBRT (12).

All patients underwent SPION-enhanced MRI simulation on the Elekta Unity MR-Linac, followed by MRI-guided adaptive liver SBRT. Patients eligible for liver transplant were included in this study. Prior liver-directed therapies, such as hepatic resections, liver SBRT, or TACE, were allowed. 

### 2.2. Workflow of SPION-Enhanced MR-Guided Liver SBRT

Patients were immobilized in a custom-molded MRI-compatible VacLoc^®^ vacuum bag (Bionix, Toledo, OH, USA). Treatment planning initiated with a free-breathing non-contrast helical computed tomography (CT) scan with 3 mm slice thickness, immediately followed by 4-D CT simulation utilizing a Siemens Somatom Sensation Open scanner (Siemens Helthineers, Erlangen, Germany) with an Anzai belt (AZ733 V, Anzai Medical, Delray Beach, Fl, USA). The tumor respiratory motion margin was assessed from the 4D-CT based on hepatic motion during the respiratory cycle. CT images from free-breathing CT were used as a study set for reference plan generation. 

Next, patients received an intravenous injection of the SPION contrast agent Ferumoxytol^®^ at 2 mg of iron per kg of body weight [16]. To minimize potential side effects associated with bolus infusion, such as hypotension and lumbar pain/leg pain (incidence from 2 to 5%), Ferumoxytol was diluted to 25–60 mL in normal saline and administered as a protracted drip infusion over 20 min, consistent with the manufacturer’s guidelines. 

A free-breathing MRI simulation was performed at 72 h after Ferumoxytol^®^ injection to allow for the imaging of the parenchymal phase of the contrast [14]. For SPION-enhanced liver imaging, a T2 3D TFE (Turbo Field Echo) MR pulse sequence in a 1.5 T Unity Philips MR scanner was employed, and the typical imaging parameters were TR/TE = 1800/205 ms, FOV = 400 × 400 mm^2^, pixel size = 1.56 × 1.56 mm^2^, image matrix = 256 × 256, thickness = 2.2 mm, and flip angle = 8°. A navigator window was set at the liver dome scout (1/3 into the lung and 2/3 into the liver). 

Gross tumor volume (GTV) was delineated via post-SPION MRI. Non-contrast CT and SPION-MR simulation images were rigidly co-registered with MIM software and manually adjusted to ensure adequate contouring of the OARs to account for positional shifts. An internal target volume (ITV) was created with an added tumor respiratory motion margin determined by 4D-CT. 

**Figure 2:**
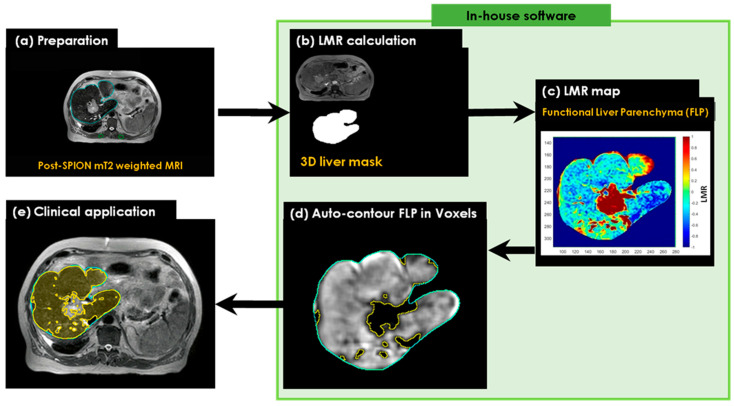
The workflow of the SPION-aided liver SBRT planning technique involves the following steps: (**a**) preparation of the post-SPION T2-weighted image set with manually defined liver contours (cyan) and paraspinal muscle contours (green); (**b**) extraction of the liver mask from the post-SPION image set; (**c**) calculation of the liver-to-average-muscle signal ratio (LMR) on the extracted liver mask and the generation of a liver function map by applying a threshold to the LMR on the post-SPION LMR map; (**d**) auto-contouring the FLV (colored area in yellow); and (**e**) overlaying the contour of the FLV on the liver image for further sparing during MRI-guided radiotherapy planning.

An automated contouring algorithm was generated for the delineation and guided avoidance of the liver’s best functional volumes. This was achieved using 1.5 T MRI-Linac-based detection and the quantification of liver parenchyma SPION iron content, based on a liver-to-muscle signal intensity ratio threshold approach [20]. T2-MR image sets were acquired before and after an intravenous injection of SPIONs. The two sets of pre- and post-SPION T2-MR image sets were transferred to MiM software (v7.0.6, MIM Software Inc, Cleveland, OH, USA), and the liver and paraspinal muscle contours on the post-SPION T2-MR image sets were manually delineated for liver SBRT planning (Figure 2a). The 3D liver contours were extracted by binary masking for the voxel-wise 3D liver volumes in both sets (Figure 2b). Next, we calculated the liver-to-muscle signal intensity ratio (LMR) and defined functional liver volume by applying a threshold of LMR (Figure 2c) and an auto-contoured FLV (see Figure 2d). Finally, we utilized our in-house software for the auto-contouring of FLV (Figure 2e) to enhance conformal avoidance during online plan adaptations on the MR-Linac. 

### 2.3. Treatment Planning

Optimized plans for Step-and-Shoot Intensity-Modulated Radiotherapy (SS-IMRT) were designed using multiple coplanar beam arrangements. The planned target volume (PTV) included the ITV plus a 0–0.5 cm margin. All patients completed liver SBRT to a median dose of 48 Gy (range 36–54 Gy) delivered in 1–5 fractions, prescribed to the isodose line encompassing PTV (generally 90% isodose line), allowing up to 20% higher dose to the target volume (10% on average). Dose per fraction varied based on tumor size, location, and normal tissue tolerance. The delineation of the tumor, functional hepatic parenchyma, and OARs was performed in Monaco, V.5.4.

Given the population’s pre-existing liver conditions, hepatic dose constraints were imposed exclusively on residual functional liver volumes defined by SPION-MRI, as previously reported [21]. 

Dose limits to hepatic parenchyma were set such that at least 30% of the predicted liver volume by MR functional imaging received ≤ 18 Gy in five fractions, ≤17 Gy in four fractions, or ≤16 Gy in three fractions. The mean dose to the functional liver volume was set to be 16 Gy. Other constraints included stomach V25 <10 cc (max < 30 Gy) and small bowel V20 < 20 cc (max < 30 Gy), where V20 and V25 are the corresponding organ volumes receiving at least 20 or 25 Gy, respectively. If radiation dose constraints for residual functionally active hepatic parenchyma or any OAR could not be met, the PTV dose was relaxed accordingly.

Daily MR image sets during per-fraction MR-SPION guided liver SBRT were acquired for online treatment plan adaptation to the per-fraction anatomical changes in the shape (ATS) of the GTV and OARs. Since the contrast effect of Feraheme from the day of injection was maintained throughout the entire treatment course, no additional administration of the drug was required. The adapted plan was evaluated by the treating physician and approved after the completion of online quality assurance (QA) checks. During dose delivery, cine MR imaging was obtained for the real-time visualization of PTV in relation to a pre-defined OAR structure set. After the completion of treatment, a “post-treatment” T2 scan was acquired. 

### 2.4. Outcome Data 

All patients were monitored for toxicity monthly for the first three months, followed by assessments every three months. These included clinical examinations, multiphase contrast-enhanced liver MRI or CT scans, and laboratory tests (complete metabolic panel, coagulation profile, and complete blood count). To more accurately evaluate treatment-related hepatic toxicity in patients with cirrhosis, we applied the Mean Model of End-Stage Liver Disease with a sodium component (MELD-Na) scoring system. Acute and long-term toxicities were graded according to the Common Terminology Criteria for Adverse Events (CTCAE) v4.0. Local response was assessed every three to four months after SBRT completion using contrast-enhanced multiphase liver MRI or CT. Local control (LC) was defined as the absence of tumor radiographic progression within or at the PTV margin. New liver lesions arising outside the PTV were classified as intrahepatic progression. Actuarial LC and overall survival (OS) curves were generated using the Kaplan–Meier method.

## 3. Results

### 3.1. Patient Demographics 

Patient demographics and tumor characteristics are presented in Table 1 and Table 2.

All patients completed SPION-enhanced MRI-guided treatment planning on an MR-Linac followed by per-fraction adaptive planning during SBRT delivery at a median dose of 48 Gy (range 36–54 Gy) delivered in 1–5 fractions. Patients had a mean cumulative tumor max diameter of 4.7 cm (range, 1–13 cm). 

Twelve liver-transplant-eligible patients with HCC and hepatic cirrhosis completed SBRT for tumor downsizing or as a bridging therapy, and eight of them received an orthotopic liver transplant (OLT), with a median time to transplant of 8 months (range, 3–15). 

### 3.2. SPION-Enhanced MRI-Guided Liver SBRT: Dosimetry Comparison

**Figure 3:**
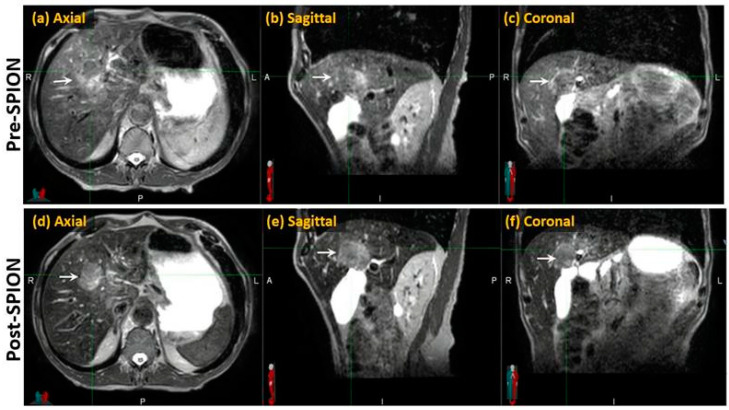
Pre- and post-SPION MR images of HCC (arrows) in the first and second row, respectively.

Compared to the pre-SPION image sets, SPIONs improved tumor visibility in the post-SPION images. The tumor boundary is clearly shown in the post-SPION images across all primary and metastatic liver tumors (Figure 3d–f), with negatively enhanced liver parenchyma. 

**Figure 4:**
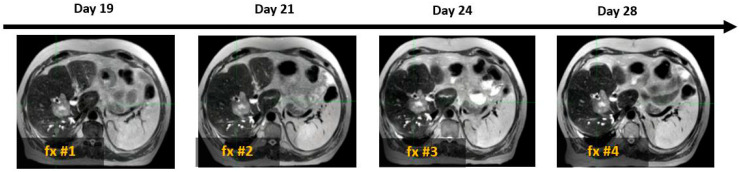
Example of four daily MR image sets used for online treatment plan adaptation targeting solitary colon cancer metastasis (white arrow) with SBRT (50 Gy in 4 fractions). Days 19, 21, 24, and 28 indicate the time elapsed since Ferumoxytol^®^ injection, corresponding to sequential SBRT fractionation (fx).

As illustrated in Figure 4, SPIONs ensured consistent tumor visibility throughout the entire treatment course of the MR-guided liver SBRT, which had a median duration of 15 days (ranging from 5 to 28 days).

### 3.3. Functional Treatment Planning

Volumes of functional liver parenchyma (FLVs) were identified by SPIO-MRL as T2 W hypointense volumes, representing hepatic Feraheme-loaded Kupffer cell masses in proportion to hepatic vascular perfusion. Contoured FLVs were used for conformal avoidance during the radiation beam placement process using the Monaco-5.4 treatment planning system. Liver dose constraints were imposed exclusively on FLVs defined by SPION-MRL. 

**Figure 5:**
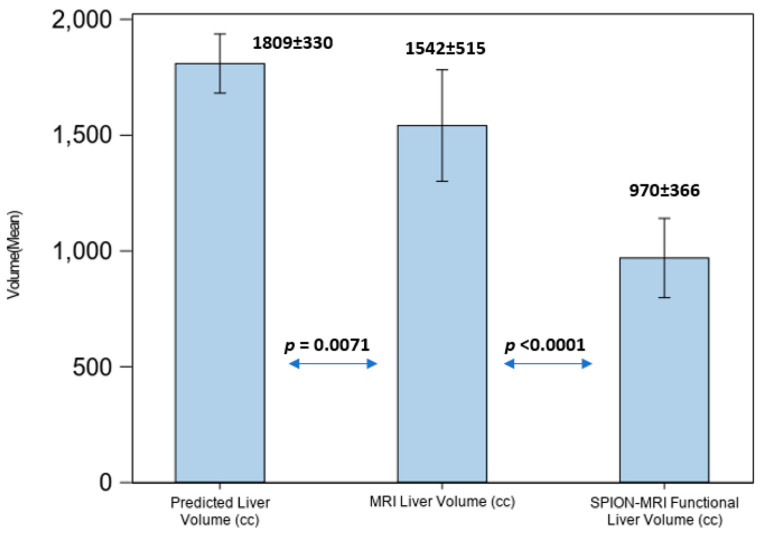
Liver volume comparison in patients with hepatic cirrhosis.

Due to fluctuations in liver volume caused by advanced cirrhosis and prior liver-directed therapies, dose limits for the hepatic parenchyma were established. Specifically, at least 30% of the predicted (calculated) normal liver volume, as defined by SPION-MR functional imaging, received a maximum dose 16 Gy, 18 Gy, or 19 Gy from SBRT, delivered in four, five, or six fractions, respectively. In patients with HCC and hepatic cirrhosis we observed reduction in the amount of functional liver volumes on SPION-MRL by 53% as percentage of predicted liver volume, but no such difference was observed for non-HCC patients with preserved liver function and morphology (Figure 5). 

**Figure 6:**
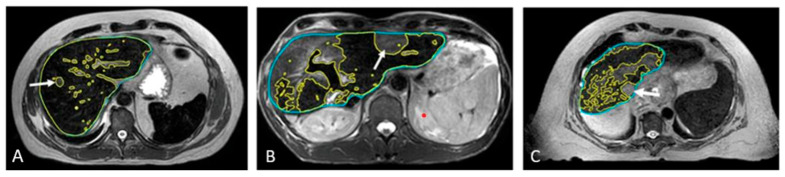
Automated contouring of functional (yellow) and anatomic (blue) liver volumes in three patients: (**A**) patient with solitary liver metastasis and normal liver function, (**B**) patient with solitary hepatic metastasis and prior liver-directed therapies, and (**C**) patient with HCC, Child–Pugh B cirrhosis, and prior SBRT (**C**). Tumors are marked with arrows.

Figure 6 presents the SPION-enhanced MR imaging of FLV in three patients: (A) patient with small colon cancer liver metastasis, with normal liver function and morphology; (B) patient with colon cancer liver metastasis and history of prior hepatectomy, radiofrequency ablation, and prior liver SBRT; and (C) patient with Child–Pugh B cirrhosis and prior SBRT to hepatic segments 2 and 8, performed a year before reirradiation with current SBRT. In the noncirrhotic patient (A), SPION distribution across the liver parenchyma was diffuse and homogeneous, with T2W imaging showing FLV closely matching the liver volume on the non-contrast MRI scan and the predicted individual liver volume. In the second patient (B) with an intrahepatic recurrence of colon cancer metastasis, FLV closely matched the individual predicted liver volume, despite the hypertrophy of healthy liver parenchyma due to prior liver-directed therapies. In contrast, patient (C) with advanced cirrhosis and prior liver-directed therapy showed a marked sequestration and retraction of FLV in the SPION-MRI. As a result, the residual FLV was significantly smaller than the anatomic liver volume from non-contrast MRI, representing only 40% of the predicted liver volume.

**Figure 7:**
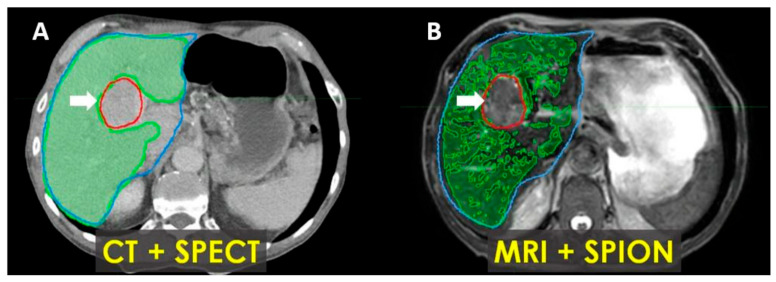
Patient with Child–Pugh A liver cirrhosis: SPECT-CT (**A**) and MRI-SPION (**B**) imaging of tumor (arrow) and functional liver volumes (FLVs) (green). Anatomic liver volume delineated in blue. There is an estimated 20% loss of functional hepatic parenchyma, with more accurate tumor delineation defined via MRI-SPION compared to SPECT/CT.

In patients with Child–Pugh A-6 cirrhosis, functional treatment planning using SPION-enhanced MRI-Linac demonstrates superior performance compared to SPECT-CT-based treatment planning, as illustrated in Figure 7. 

For all patients with hepatic cirrhosis in our study, the liver volume defined by MRI was an overrepresentation of the volumes of functionally active liver parenchyma by a factor of 1.8 on average. Compared with predicted LV, cirrhotic patients demonstrated an average FLV loss of 53% (*p* < 0.0001) based on repeated ANOVA results.

**Figure 8:**
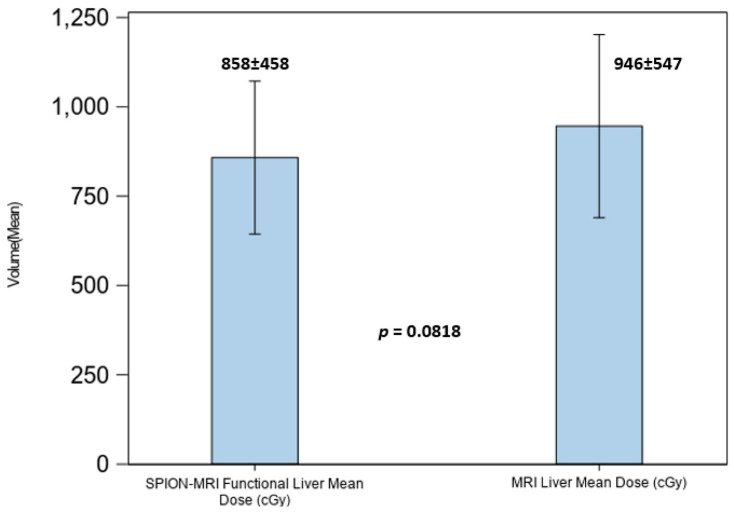
Mean dose to the anatomic liver volume in MRI (right) and functional liver volume in SPION-enhanced MRI (left).

Despite the significant loss of FLV in patients with hepatic cirrhosis, optimal radiation beam placement with SS-IMRT optimization and per-fraction plan adaptations to the SPION-enhanced MR-Linac treatment permitted the effective avoidance of residual functional hepatic parenchyma from the above threshold irradiation. This resulted in lowering the mean dose to residual FLV to 8.6 Gy ± 4.58 Gy, exceeding our liver dosimetric objective (Figure 8). 

### 3.4. Treatment Outcome

At a median follow-up of 23 months (range, 3–40 months), in-field local tumor control for the entire group was 100%, and the overall 2-year survival rate was 65% (100% after OLT). Among the metastatic group, five of nine patients and four in the HCC group received an additional course of SBRT for isolated intrahepatic recurrence outside the treated field. Two of nine patients (22%) with hepatic oligometastases experienced continuous intrahepatic and systemic cancer progression leading to death. Among eleven transplant-ineligible patients with HCC and cirrhosis (CP-A: four patients; CP-B: seven patients) the median survival was 23 months (range, 3–37 months) with cirrhosis-related complications being the leading cause of death. 

Twelve liver-transplant-eligible patients underwent SPION-enhanced MRI-guided functional treatment planning on an MR-Linac and received liver SBRT to a mean dose of 43.6 Gy (range: 26–50 Gy) administered over 1–5 fractions before orthotopic liver transplantation (OLT). Four patients were taken off the transplant list due to the following: multifocal intrahepatic progression (2), the development of a second malignancy (1), and one patient with a controlled tumor and compensated cirrhosis withdrew from the liver transplant pathway. 

Eight patients successfully underwent OLT at a median of 8 months (range 3–15) after SBRT completion. Patients with multifocal HCC received either simultaneous or sequential SBRT to lesions larger than 2 cm. Of these patients, five met the Milan criteria for transplantation and underwent SBRT as a bridging therapy before OLT, while two did not meet the criteria and received SBRT for tumor downsizing prior to transplantation. 

On pathologic examination of the explanted livers, four cases (50%) demonstrated a complete pathologic response, while another four patients showed varying degrees of tumor size reduction. Notably, none of the patients experienced tumor progression. Overall, the median viable tumor size in explanted livers was 1.3 cm (0–2.4 cm), which was significantly smaller than the original tumor size (mean: 3.21 cm; range 2.1–6.6 cm) as measured via MRI before the SBRT (*p* < 0.001). Additionally, the median wait time for a liver transplant was 8.25 months (range, 3–15 months).

**Figure 9:**
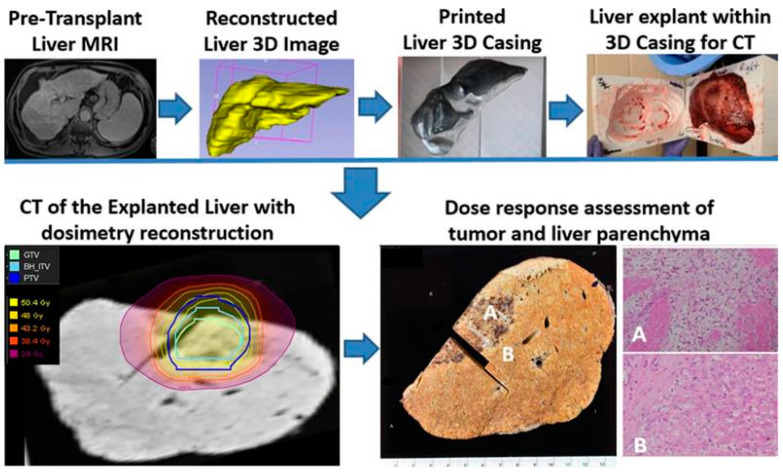
Study schema for the assessment of tumor and liver parenchymal dose–response relationship in a patient who completed liver SBRT (50 Gy in 5 fractions) as a bridge to transplant on SPION-enhanced MR-LINAC. A photograph of the cut section of the explained native liver shows a necrotic tumor and surrounding hepatic parenchyma with fibrosis, congestion, and venous thrombosis approximately within the 30-Gy isodose envelope (**A**). In contrast, the liver outside the 30-Gy isodose line appears normal (**B**). The corresponding microphotograph (**A**) reveals histopathologic features of radiation-induced liver disease, including the accumulation of pigmented macrophages, collagen deposition, venous thrombosis and congested sinusoids. In comparison, microphotograph (**B**) outside a 30-Gy isodose line shows Child-Pugh A cirrhotic liver without radiation-induced histopathological changes.

One patient in our series underwent liver transplant at 3 months after the completion of planned SBRT as a bridge to transplant. The explanted specimen was examined using our unique 3D conformal fusion approach, in which the SBRT dosimetry data on a treatment planning CT image set were overlaid with a corresponding pre-transplant MRI and co-registered with a CT image of the explanted liver and with multiple coplanar 2-D microphotographs of the pathology sections across the tumor and hepatic parenchyma. Three-dimensional printing of the liver casing from the pre-transplant liver MRI was required to restore the anatomic shape and orientation of the explanted liver for accurate image co-registration. Figure 9 represents our single-case 3D conformal fusion technique for the assessment of the tumor and the hepatic parenchymal dose–response relationship in a transplant patient. 

We observed morphologic features of radiation-induced liver damage within the planned target volume (PTV, marked by the blue line) exposed to 50 Gy. Outside the 25 Gy isodose envelope, the liver parenchyma showed complete regeneration, with a sharp demarcation visible on the gross specimen and corresponding microphotographs (Figure 9A,B). 

None of the patients received radiation doses exceeding FLV tolerance. No cases of classic or non-classic RILD (defined as rapid elevation of bilirubin or liver enzymes >5× upper limit of normal) were observed in the entire cohort within 3 months post-SBRT. Three incidences of transient grade 2 gastrointestinal toxicity were observed (nausea/vomiting controlled with medication). The most common toxicity was grade ≤2 fatigue (45% grade 1, 15% grade 2, and 0% grade 3) which did not correlate with the amount of FLV loss or the severity of hepatic cirrhosis based on the MELD-Na score.

No accelerated (within 6 months) CP class migration from A to B or from B to C was observed based on our calculations and an independent assessment by the hepatology and transplant surgery teams. 

Additionally, no statistically significant differences were observed in mean MELD-Na scores at 3 to 4 months, 6 months, or 12 months post-SBRT. 

## 4. Discussion

Clinicians treating patients with primary and metastatic liver tumors currently have several curative and palliative options at their disposal, including surgery (resection or transplantation), radiotherapy, thermal ablation, transarterial techniques (bland, chemo-, or radio-embolization), and systemic therapies (cytotoxic, targeted, or immune-based treatments) [22]. These treatment modalities are frequently employed in combination to optimize patient outcomes. However, underlying liver disease, including cirrhosis, hepatitis, and non-alcoholic steatohepatitis, often limits treatment feasibility and increases the risk of complications. Additionally, prior liver-directed or systemic therapies may induce varying degrees of parenchymal injury, further compromising hepatic function. The impact of these injuries is multifaceted, including direct hepatic damage from hepatectomy, radiation-induced liver disease (RILD), chemotherapy-associated sinusoidal obstruction syndrome, and ischemic damage from embolization. Given these challenges, improving the quality and precision of liver-directed therapies is essential to preserving adjacent functional parenchyma while maximizing tumor control.

Stereotactic body radiotherapy (SBRT) has emerged as a highly effective curative treatment for primary and metastatic liver tumors, including as a bridge-to-transplant therapy for hepatocellular carcinoma (HCC) patients with cirrhosis [23,24]. However, liver SBRT can be associated with an elevated risk of RILD, particularly in patients with underlying hepatic dysfunction, which can significantly impact post-treatment survival [25,26,27]. Since the overall survival of these patients is closely tied to residual hepatic functional reserve, there is an important unmet need to develop a clinically applicable precision-based liver SBRT planning technique with enhanced visualization and a guided avoidance of residual functional liver parenchyma.

We have previously developed a novel visualization and guided avoidance planning strategy for liver SBRT, using single-photon emission computerized tomography (SPECT) to highlight residual functionally active hepatic parenchyma in proportion with the severity of hepatic cirrhosis through the uptake of ^99m^Tc–sulfur colloid by hepatic sinusoidal macrophages or Kupffer cells (KC) [21]. Kupffer cells are highly radiosensitive, play a pivotal role in the initiation and development of RILD [28,29,30,31], and may accelerate the progression of liver fibrosis/cirrhosis [32]. Our use of an SPECT-CT-based functional treatment planning approach for patients with HCC and advanced hepatic cirrhosis undergoing bridge-to-transplant liver SBRT demonstrated high effectiveness and low toxicity with no cases of RILD [21,33]. However, the physical and technical performance characteristics of nuclear medicine techniques like SPECT-CT limit their quantitative assessment and cause image degradation. These limitations have led us to explore the integration of rapidly evolving MRI-linac hybrid technology into our planning and treatment protocols. We have begun to explore the combination of MR-linac with Ferumoxytol, an FDA-approved super-paramagnetic iron oxide nanoparticle (SPION) that was developed as a pharmaceutical therapy for anemia, but is increasingly utilized as an alternative contrast agent for liver MRI [14,15,16]. In addition to its excellent safety profile in patients with impaired kidney function [13], this agent can be used to assess a healthy liver based upon its uptake by hepatic Kupffer cells (KC), which infiltrate hepatic parenchyma but are diminished or absent in tumors [16,19]. Due to its selective uptake by hepatic Kupffer cells, Ferumoxytol produces strong, uniform T2, and T2* relaxation effects in functional liver parenchyma, while sparing tumor regions that lack Kupffer cells. 

Early results incorporating functional treatment planning using SPION-enhanced MRI-Linac for liver SBRT demonstrated the effectiveness of this approach for the quantitative characterization of functional liver parenchyma and enhanced tumor visualization [34]. Our findings also suggest that it outperforms SPECT-CT-based treatment planning in cirrhotic patients undergoing liver SBRT (Figure 7). 

Compared to pre-SPION image sets, SPION significantly enhanced tumor visibility in post-SPION images of HCC and hepatic metastases (Figure 3 and Figure 4). Tumor boundaries were clearly delineated against the negatively enhanced functional liver parenchyma in the post-SPION images (Figure 3, Figure 4 and Figure 6). 

The prolonged retention of SPION contrast within the hepatic parenchyma enabled per-fraction treatment plan adaptation using SPION-enhanced MR imaging of the tumor and functional hepatic parenchyma throughout the entire treatment course, which had a median duration of 15 days (ranging from 5 to 28 days), without the need for repeated contrast administration.

As most of our patients had pre-existing liver conditions, hepatic dose constraints were imposed exclusively on residual functional liver volumes defined by SPION-MRI. For patients with hepatic cirrhosis in our study, the liver volume defined by MRI was an overrepresentation of the volumes of functionally active liver parenchyma by a factor of 1.8 on average. Radiation dose limits to functional hepatic parenchyma were set to ensure that at least 35% of the predicted liver volume, rather than anatomic liver volume defined by MRI or CT, was spared. This approach maximizes the guided avoidance of residual functionally active hepatic parenchyma and is detailed in our prior report [21]. Despite a significant loss of FLV, radiation beam placement with SS-IMRT optimized planning and per-fraction plan adaptations to SPION-enhanced MR-Linac treatments, permitting the effective avoidance of residual functional hepatic parenchyma with regard to the above threshold irradiation. This led to a reduction in the mean dose to the residual functional liver volume to 8.6 Gy ± 4.58 Gy, surpassing our dosimetric objective of 16 Gy (Figure 8). 

At a mean dose of 48 Gy (range 36–54 Gy) delivered in 1–5 fractions and a median follow-up of 23 months (range, 3–40 months), our in-field local tumor control was 100%, with an overall survival of 65% (100% after OLT in selected patients with HCC), similar to the rates previously reported [35,36]. Nearly all patients in our study had prior liver-directed therapies, including patients with hepatic metastases who had completed prior liver SBRT (6), hepatic resection (2), or a combination of both (4). Seven patients in the HCC subgroup had completed prior TACE, and 12 patients had had prior liver SBRT (Table 1). Notably, 78% of HCC patients had Child–Pugh B hepatic cirrhosis at a mean MELD-Na score of 13.9 (range, 8–21), and 19 patients in the HCC subgroup had completed either prior TACE (7) or prior liver SBRT (12). Despite the high-risk patient cohort in our study, the observed toxicity was comparable to or lower than expected based on other reports [35,36,37,38]. We believe that the implementation of the guided avoidance of residual FLV during treatment planning with a SPION-MR-Linac may contribute to the amelioration of radiation-induced liver disease in patients with advanced hepatic cirrhosis, as evidenced by the stable MELD-Na scores over 6 months from SBRT completion and the absence of RILD.

Furthermore, SPION-enhanced MRI-guided SBRT demonstrated promising outcomes as a bridging therapy for liver transplantation. Among transplant-eligible patients, eight successfully underwent orthotopic liver transplantation (OLT) following SBRT, with no cases of tumor progression. The histopathological analysis of explanted livers revealed complete pathologic response in 50% of cases, underscoring the potential of SPION-enhanced MRI-SBRT as a highly effective pre-transplant treatment strategy. Additionally, our novel 3D conformal fusion approach, integrating SBRT dosimetry with pre-transplant MRI and pathologic examination, provided valuable insights into the dose–response relationships and potential hepatoprotective effects of Ferumoxytol. We observed no classic morphologic features of radiation-induced liver damage within the planned target volume (PTV—blue line) exposed to 50 Gy, with complete regeneration of the liver parenchyma outside the 25 Gy isodose envelope (Figure 9A,B). This latter finding may provide early evidence for the potential cyto-protective effects of Ferumoxytol [39], conferring radio-resistance or an enhanced regeneration of normal liver parenchyma exposed to lower radiation doses. 

The main limitation of this study is its relatively small sample size, which may not be sufficient to establish standardized protocols for SPION-enhanced MR-guided radiotherapy. Further validation in a larger, multicenter Phase II study is needed. Additionally, future research should investigate the potential cytoprotective properties of SPION-Feraheme in mitigating radiation-induced tissue damage and promoting normal tissue regeneration. 

## 5. Conclusions

This study represents the first direct assessment of the impact of the superparamagnetic iron oxide nanoparticle (SPION) contrast agent Ferumoxytol^®^ on tumor visibility and functional liver mapping during per-fraction adaptive radiation therapy planning using the 1.5 T Elekta Unity MR-Linac system. Our findings demonstrate that SPION-enhanced MRI provides consistent tumor visualization throughout SBRT, thereby facilitating precise tumor targeting with a conformal avoidance of functional liver parenchyma.

A key observation in our study is that the liver SBRT planning approach was not uniform, but rather highly personalized. Radiation dose constraints were tailored exclusively to each patient’s residual functionally active volumes of hepatic parenchyma, accounting for pre-existing liver conditions such as cirrhosis or prior liver-directed therapies. This novel approach is particularly beneficial for patients with HCC and hepatic cirrhosis requiring bridging SBRT with liver function preservation while awaiting liver transplantation. Furthermore, this study underscores the potential of ferumoxytol-enhanced MRI to overcome the limitations of conventional liver imaging modalities, particularly in patients with kidney insufficiency, where gadolinium-based contrast agents are contraindicated. 

Overall, our results suggest that SPION-enhanced MRI has significant implications for advancing personalized liver cancer radiotherapy, reducing off-target hepatic toxicity, and improving clinical outcomes in patients with compromised liver function. Future research should focus on validating these findings in larger cohorts and exploring the longitudinal impact of SPION-enhanced imaging on tumor and hepatic parenchyma response assessments and treatment adaptations.

## Figures and Tables

**Table 1:** Patient characteristics.

Patients with Metastases (*n* = 9)	
Median age	55 years (50–72)
Timing of metastases - Synchronous (at diagnosis) - Metachronous	2 7
Origin - Colorectal - Breast	6 3
Prior Liver-directed therapies: SBRT Liver resection Combination of both	8 (88%) 6 (66%) 2 (22%) 4 (44%)
Extrahepatic metastatic sites - No - Yes	7 (78%) 2 (22%)
Patients with HCC (*n* = 23)	
Median Age	70 years (53–87)
Child-Pugh Class/Score A/5.3 (5–6) B/7.5 (7–10)	5 (12%) 18 (78%)
MELD Score ≤9 10–12 13–15 >15	7 (30%) 9 (40%) 3 (13%) 4 (17%)
Prior liver-directed therapies: TACE SBRT Combination	7 (30%) 12 (52%) 2 (9%)

**Table 2:** Tumor characteristics.

Characteristics	HCC (*n* = 23)	Metastases (*n* = 9)
Number of lesions treated Tumor diameter (cm)	26	14
1–2	5	4
2–4	11	7
5–6 >6	6 4	2 1
Dose (Gy) Number of fractions	41.8 ± 8.1 4.1 ± 1.2	49.6 ± 4.1 4.3 ± 0.9

## Data Availability

The original contributions presented in the study are included in the article; further inquiries can be directed to the corresponding author(s).
